# Supplementary material for: The interactome of intact mitochondria by cross-linking mass spectrometry provides evidence for coexisting respiratory supercomplexes
Source: Mol Cell Proteomics. 2017 Dec 8;17(2):216–32. doi: 10.1074/mcp.RA117.000470 (PMC5795388; doi:10.1074/mcp.RA117.000470)
Supplement: Supplemental Data [file supp_RA117.000470_133922_0_supp_23978_fzffwf.zip › spectra_annotation/mito_spectra_annotation/282-1-5-1-3-1.pdf]

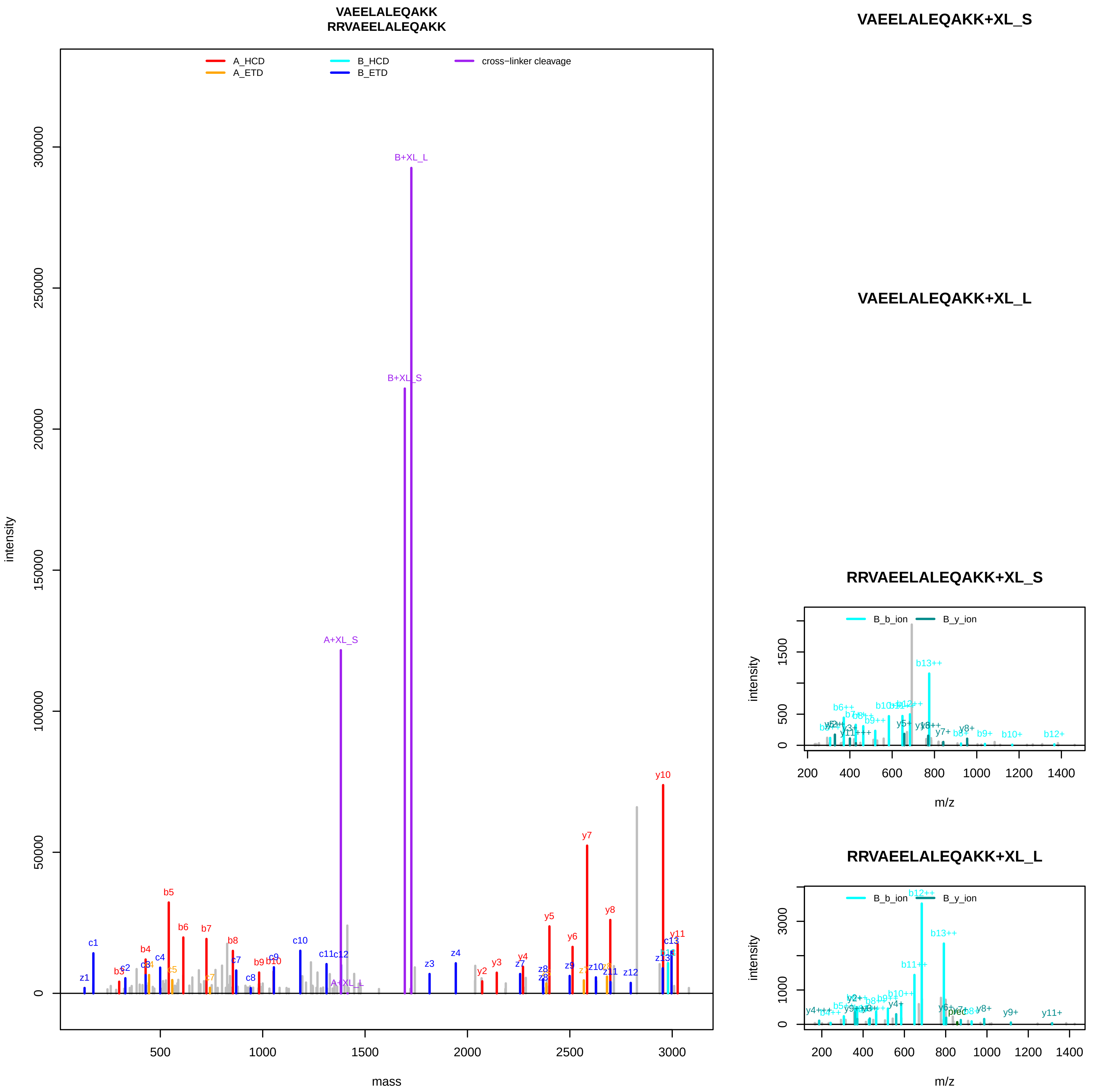
VAEELALEQAKK
RRVAEELALEQAKK
A_HCD
A_ETD
B_HCD
B_ETD
cross−linker cleavage
B+XL_L
B+XL_S
A+XL_S
A+XL_L
y10
y7
y8
y5
y6
y11
b5
b6
b7
b8
b4
b3
b9
b10
c1
c2
c3
c4
c5
c7
c8
c9
c10
c11
c12
c13
z1
z3
z4
z7
z8
z9
z10
z11
z12
z13
y2
y3
y4
intensity
mass
0
50000
100000
150000
200000
250000
300000
500
1000
1500
2000
2500
3000
VAEELALEQAKK+XL_S
VAEELALEQAKK+XL_L
RRVAEELALEQAKK+XL_S
B_b_ion
B_y_ion
b13++
b12++
b6++
b9++
b10+
b12+
b9+
y8+
y7+
intensity
m/z
0
500
1500
200
400
600
800
1000
1200
1400
RRVAEELALEQAKK+XL_L
B_b_ion
B_y_ion
b12++
b13++
b11++
b10++
y4+
y8+
y9+
y11+
intensity
m/z
0
1000
2000
3000
200
400
600
800
1000
1200
1400
